# Post-operative neutrophil-to-lymphocyte ratio and outcome after thrombectomy in acute ischemic stroke

**DOI:** 10.3389/fneur.2022.990209

**Published:** 2022-09-28

**Authors:** Shen-Jie Li, Shan-Shan Cao, Pei-Sheng Huang, Xin Nie, Yang Fu, Jian-Ren Liu

**Affiliations:** ^1^Department of Neurology, Stroke Center, Shanghai Ninth People's Hospital, Shanghai Jiao Tong University School of Medicine, Shanghai, China; ^2^Biostatistics Office of Clinical Research Unit, Shanghai Ninth People's Hospital, Shanghai Jiao Tong University School of Medicine, Shanghai, China

**Keywords:** ischemic stroke, mechanical thrombectomy, neutrophil, lymphocyte, outcome

## Abstract

**Background:**

Neutrophil to lymphocyte ratio (NLR) is a novel inflammatory marker to predict adverse cardiovascular events. However, there is a lack of data on hemorrhagic transformation (HT) and neurological outcome after mechanical thrombectomy in acute ischemic stroke (AIS). We investigated whether NLR before and after thrombectomy for patients with AIS was associated with HT and neurological outcomes.

**Methods:**

We performed a retrospective analysis of consecutive patients with anterior circulation AIS who underwent thrombectomy. HT was evaluated by CT within 24 h after thrombectomy. Clinical data had been collected retrospectively; laboratory data were extracted from our electronic hospital information system. NLR was obtained at admission (NLR1) and immediately after thrombectomy (NLR2). The main outcomes were post-interventional intracranial hemorrhage and unfavorable functional status (modified Rankin scale scores of 3–6) 3 months post-stroke.

**Results:**

A total of 258 patients with AIS, according to the NIHSS (median 14), were included. NLR2 was higher in patients who developed HT after thrombectomy and unfavorable neurological outcomes 3 months post-stroke (*p* < 0.001) than in those without HT or favorable outcomes, even after correction for co-factors [Odds Ratio (OR) 1.35 for HT, 95% confidence interval (CI)1.16–1.57, *p* < 0.001, and 1.85 for unfavorable outcome, 95%CI 1.57–2.17, *p* < 0.001]. The optimal cutoff value for the NLR2 as an indicator for auxiliary diagnosis of HT and the unfavorable outcome was 8.4 and 8.8, respectively.

**Conclusion:**

NLR immediately after thrombectomy is a readily available biomarker of HT and neurological outcomes in patients with AIS.

## Introduction

Acute ischemic stroke (AIS) is a common disease that affects elderly people (1). A randomized controlled trial confirms the improved reperfusion, early neurologic recovery, and functional outcome of thrombectomy compared to intravenous thrombolysis (IVT) (2). However, hemorrhagic transformation (HT) is a secondary intracranial hemorrhage after thrombectomy, with an incidence of about 10%. It is a major complication of thrombectomy and AIS in the acute phase (3), which often indicates a poor prognosis (4). Therefore, to improve the effectiveness of thrombectomy in clinical practice, it is of great significance to identify the risk factors of HT after thrombectomy.

Inflammation and immune responses run through all stages of cerebral ischemia and stroke progression, and when an ischemic stroke occurs, brain tissue releases pro-inflammatory chemokines, triggering strong inflammatory responses (5). Ischemic stroke reperfusion occurs after an endovascular procedure, and the damaged tissue is reoxygenated (6), resulting in the accelerated production of reactive oxygen species (ROS) and reactive nitrogen species. Inflammation caused by increased free radicals triggers the accumulation of inflammatory cells in the ischemic area. Interactions between endothelial cells and inflammatory cells lead to the release of many cytokines and amplify ischemic injury through reperfusion. Circulating neutrophils enter injured brain regions shortly after ischemia and then participate in disrupting the blood–brain barrier (BBB) and increasing tissue damage (7, 8). The greater the number of neutrophils, the greater the enhancement of tissue damage, as they lead to the release of inflammatory mediators, ROS, and various proteolytic enzymes. Lymphopenia may reflect cortisol-related stress responses and sympathetic tone (9), which may increase pro-inflammatory cytokine production (10). NLR as a reflection of innate (neutrophil) and adaptive (lymphocyte) immune responses has been extensively studied to assess the severity of inflammation associated with systemic or local diseases. Compared with a single index, a comprehensive index has higher validity and specificity. An increased NLR level with neutrophil exaltation and lymphocyte depletion suggests an imbalance in the interplay between central and peripheral inflammation induced by stroke.

However, previous studies focused on higher preoperative NLR levels as an independent predictor of symptomatic intracerebral hemorrhage (sICH) after thrombectomy (11). Stroke-related inflammation may be more closely related to HT after thrombectomy and functional status 3 months post-stroke, and NLR can be more accurately assessed in early follow-up after thrombectomy.

In the present study, we sought to investigate the association of NLR levels immediately after thrombectomy with thrombectomy-related HT and neurological outcomes in patients with AIS.

## Methods

### Study population

This retrospective study collected the medical history and clinical examination data of 258 patients with acute anterior circulation stroke who received thrombectomy from June 2015 to December 2021 in the Department of Neurology, Shanghai Ninth People's Hospital, Shanghai Jiao Tong University School of Medicine.

**Figure 1:**
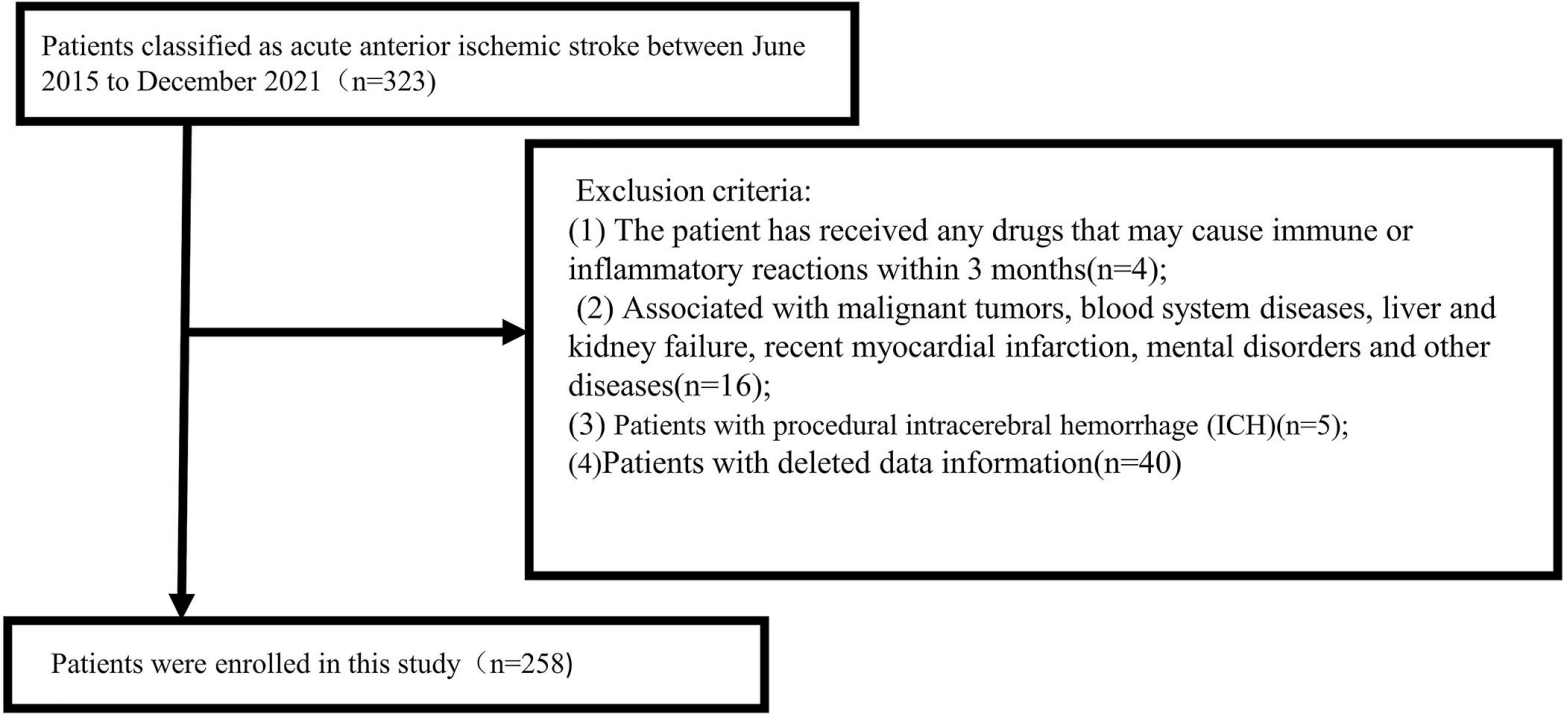
Flowchart of participant selection.

The inclusion criteria were as follows: (1) age ≥ 18 years; (2) computed tomography angiography (CTA)/magnetic resonance angiography (MRA) showing the occlusion of intracranial large arteries in the anterior circulation (intracranial carotid artery or middle cerebral artery trunk M1 or dominant M2 segment); (3) Alberta Stroke Program Early CT Score ≥ 6; and (4) the study criteria requiring preoperative and postoperative complete blood counts and laboratory data after thrombectomy immediately. The exclusion criteria were follows: (1) The patient has received any drugs that may cause immune or inflammatory reactions within 3 months; (2) The patient is associated with malignant tumors, blood system diseases, liver and kidney failure, recent myocardial infarction, and mental disorders; (3) patients with procedural intracerebral hemorrhage (ICH), i.e., ICH is clearly related to the procedure itself, such as secondary to vascular perforation, arterial dissection, or subarachnoid hemorrhage; and (4) patients with deleted data information. All inclusion and exclusion criteria were completed by two trained senior neurologists. A total of 258 patients were included in the study (Figure 1).

### Clinical assessments and laboratory parameters

The clinical data and laboratory test indexes were collected. Demographic characteristics include age and gender. Vascular risk factors include the history of hypertension, diabetes mellitus, atrial fibrillation (AF), history of stroke, coronary heart disease (CHD), smoking, and alcohol consumption. Clinical data include systolic blood pressure, diastolic blood pressure on admission, NIHSS score on admission (range 0–42, higher scores indicate more severe neurological deficit), time from onset to recanalization, time from arrival to recanalization, occlusion site, cause of stroke (TOAST classification), and Alberta Stroke Program Early CT Score (ASPECTS). Laboratory indicators include platelet count and leukocyte classification. Venous blood samples were obtained before and after thrombectomy immediately. We routinely performed detailed blood examinations before and immediately after the procedure of thrombectomy. A hematological test was collected with an EthyleneDiamine Tetraacetic Acid (EDTA) tube, and biochemical analysis was collected with a dry tube. NLR was calculated by dividing the absolute neutrophil count by the absolute lymphocyte count at each time point.

Each patient was reviewed for routine cranial CT examination within 24 h after treatment. HT was defined as bleeding observed on follow-up CT. According to the European Cooperative Acute Stroke Study (ECASS) criteria (12), HT was divided into hemorrhagic infarction (HI) and parenchymal hematoma (PH), which were further separately divided into two subtypes: HI1 type, small patch hemorrhage at the edge of the infarction; HI2 type, fusion patch hemorrhage in the infarct area, no mass effect; PH1 type: hematoma < 30% of the infarct area, the space-occupying effect is light; and PH2 type: the hematoma exceeds 30% of the infarct area, with obvious space-occupying effect. The 3-month functional prognosis was assessed according to the modified Rankin Scale (mRS). Favorable and unfavorable functional prognoses were defined as mRS ≤ 2 and mRS > 2, respectively. The time deviation of functional prognosis follow-up did not exceed 7 days.

### Treatment protocol

All the selected patients were treated by femoral artery puncture, combined with CTA, clinical manifestations, and Digital subtraction angiography (DSA) results to determine the occlusion site. Using coaxial catheter technology, the tip of the micro-catheter (Rebar 21/27, EV3, USA, or Prowler select plus, Johnson & Johnson, USA) was put at the distal end of the occluded artery under the guidance of a micro-guide wire (0.014 in Synchro-14, Stryker, U.S.), and then, the micro-guide wire was withdrawn. Subsequently, cerebral angiography *via* a micro-catheter confirmed that the micro-catheter was in the arterial lumen. The stent retriever [Solitaire FR stents (EV3, USA), Embotrap Revascularization System (Johnson & Johnson, USA), or Trevo Retriever (Stryker Neurovascular, USA)] with diameters of 4–6 mm and lengths of 15–30 mm were selected according to the diameter of the occluded blood vessels. The stent was introduced to the distal end of the occlusion through the micro-catheter and then was released. Selective angiography was performed through the intermediate catheter to observe the status of the stent, the patency of the diseased vessel, and the character and shape of the thrombus; then, negative pressure was applied to the mediate and guiding catheter while slowly withdrawing the stent and removing the thrombus. After thrombectomy, reexamination angiography was performed to observe whether the diseased vessels were opened. Recanalization of the main arteries and branches was confirmed by reexamination, indicating that the thrombus was successfully removed. The number of stent retriever passes per procedure was no more than four times. If there are no contraindications, patients within 4.5 h after the onset of the disease shall be given intravenous thrombolysis according to the guidelines, and the process of thrombectomy (i.e., bridging treatment) shall be started at the same time. We extracted angiograms after thrombectomy, and the reported angiographic results (mTICI scores) were rescored and confirmed by neuro-interventionalists who were blinded to procedures, functional outcomes, and testing. Successful reperfusion was defined as mTICI ≥ 2b, and complete reperfusion was defined as mTICI 2c/3. When recanalization of the target artery fails, the treatment team will conduct adequate evaluation and, if necessary, carry out rescue treatment, including balloon angioplasty, stent implantation, intra-arterial thrombolysis, and intra-arterial or intravenous injection of tirofiban.

### Statistical analysis

The Kolmogorov–Smirnov test was performed on all quantitative data. If the quantitative data conformed to a normal distribution, the mean ± standard deviation (*X* ± s) was used to represent the difference between the two groups. The comparison was analyzed by independent *t*-test; if the quantitative data conformed the skewed distribution, the medians and interquartile ranges (IQR) were used, and the comparison between the two groups was performed by the Mann–Whitney *U* test. The categorical data were expressed as numbers and percentage (%) of cases. The Chi-square test or Fisher's exact test was used in statistical analyses of categorical variables. Paired *t*-test was used for the NLR difference between admission and re-measurement following a normal distribution. Otherwise, the Wilcoxon signed rank sum test was used. The relation of the NLR with two endpoints was investigated using logistic regression models. For multivariate analysis, we first included age and sex (model 1) and then additionally included variables that significantly correlated with HT or unfavorable outcomes in the univariate analysis (*p* < 0.10; models 2 or 3). The receiver operating characteristic (ROC) curves were drawn to analyze the predictive value of NLR2 on the occurrence of HT and unfavorable outcomes in patients with anterior circulation AIS after thrombectomy and to establish optimal cutoff points, at which the sum of the specificity and sensitivity was the highest. A *P*-value < 0.05 was considered statistically significant. All analyses were performed using SPSS 25.0 software (IBM, Armonk, NY, United States), and figures were drawn with the use of Powerpoint software 2019 and Graphpad Prism 8.0 (GraphPad Software).

### Ethical approval

The study was approved by the Ethics Committee of Shanghai Ninth People's Hospital, Shanghai Jiao Tong University School of Medicine (No. SH9H-2020-T390-2), in accordance with Chinese legislatio; written informed consent was waived for the retrospective analysis of data collected as part of routine clinical care in these cohorts, but patients were informed that, according to Chinese legislation, they could oppose the use of their data for research purposes.

## Results

### Patient characteristics

We selected a total of 258 patients with acute ischemic stroke diagnosed with anterior circulation large artery occlusion undergoing thrombectomy who were hospitalized in the Department of Neurology, Shanghai Ninth People's Hospital, Shanghai Jiao Tong University School of Medicine from June 2015 to December 2021. Sixty-five patients were excluded due to lack of information or other reasons (Figure 1). The median age of enrolled patients was 70 (61, 79) years. There were 156 men (60.5%) and 102 women (39.5%). Among the 258 patients, 98 (38%) presented with HT on repeated cranial CT after thrombectomy (median age, 71; IQR, 61.8–79; men, 56.1%), and 143 (55%) developed unfavorable outcomes (median age, 71; IQR, 61–80; men, 60.8%). According to the presence or absence of HT or the favorable or unfavorable outcomes, the main baseline data and clinical outcomes of patients are presented in Table 1.

**Table 1:** Baseline characteristics of patients according to the presence/absence of HT and neurological outcomes at 3 months.

**Characteristics**	**Without-HT (*n* = 160)**	**With-HT (*n* = 98)**	***p***	**Favorable outcome (*n* = 115)**	**Unfavorable outcome (*n* = 143)**	***p***
**Demographics**						
Age, years, median (IQR)	70(61, 79)	71(61.8, 79)	0.854	70(62, 78)	71(61, 80)	0.722
Sex, men, %	101(63.1%)	55(56.1%)	0.264	69(60.0%)	87(60.8%)	0.891
**Risk factors**						
Smoking, %	52(32.5%)	27(27.6%)	0.403	33(28.7%)	46(32.2%)	0.548
Drinking, %	29(18.1%)	14(14.3%)	0.422	20(17.4%)	23(16.1%)	0.779
Hypertension, %	95(59.4%)	62(63.3%)	0.534	65(56.5%)	92(64.3%)	0.201
Diabetes, %	38(23.8%)	21(21.4%)	0.667	22(19.1%)	37(25.9%)	0.200
Stroke history, %	16(10.0%)	15(15.3%)	0.210	10(8.7%)	21(14.7%)	0.136
Atrial fibrillation, %	74(46.3%)	50(51.0%)	0.485	52(45.2%)	72(50.5%)	0.450
Coronary heart disease, %	66(41.3%)	47(48.0%)	0.292	47(40.9%)	66(46.2%)	0.395
**Clinical data**						
Systolic BP (mmHg), median (IQR)	145.5(128, 162.8)	142.5(128, 172.3)	0.618	142(127, 160)	146(130, 170)	0.205
Diastolic BP (mmHg), median (IQR)	80(71.3, 90)	80(73, 95.8)	0.240	80(72, 90)	80(73, 94)	0.230
Admission NIHSS, median (IQR)	14(9,19)	14(11,18)	0.463	13(8,18)	15(12, 19)	**0.003**
Time from onset to recanalization (minutes), median (IQR)	414(281.5, 600)	387.5(307.8, 607)	0.962	390(270, 572)	410(304, 628)	0.442
Time from arrival to recanalization (minutes), median (IQR)	206.5(148.3, 273.8)	219.5(179, 275)	0.090	218(155, 270)	209(167, 275)	0.938
Prior IVT, %	57(35.6%)	31(31.3%)	0.511	40(34.8%)	48(33.6%)	0.838
ASPECTS, median (IQR)	9(9,9)	7 (6,8)	**< 0.001**	9(8,9)	8(7,9)	**< 0.001**
Recanalization (mTICI ≥2b), %	142(88.8%)	23(23.5%)	**< 0.001**	95(82.6%)	70(49.0%)	**< 0.001**
Procedural modes			0.717			0.472
Stent retriever only	65(40.6%)	44(44.9%)		51(44.3%)	58(40.6%)	
Stent retriever with rescue therapy	53(33.1%)	28(28.6%)		38(33.0%)	43(30.1%)	
Other modes without stent retriever	42(26.3%)	26(26.5%)		26(22.6%)	42(29.4%)	
Occlusion site, %			0.084			**< 0.001**
ICA	56(35.0%)	22(22.4%)		49(42.6%)	29(20.3%)	
MCA	77(48.1%)	53(54.1%)		49(42.6%)	81(56.6%)	
Tandem occlusion	27(16.9%)	23(23.5%)		17(14.8%)	33(23.1%)	
Cause (TOAST), %			0.082			0.255
LAA	83(51.9%)	37(37.8%)		60(52.2%)	60(42.0%)	
CE	44(27.5%)	33(33.7%)		30(26.1%)	47(32.9%)	
Other	33(20.6%)	28(28.6%)		25(21.7%)	36(25.2%)	
**Laboratory data**						
Blood glucose, mmol/L, median (IQR)	6.9(5.9, 8.7)	7.2(5.6, 9.2)	0.935	6.7(5.6, 8.1)	7.2(5.8, 9.5)	0.120
Platelet1, × 10^9^/L, median (IQR)	194(159.3, 235.5)	190.5(156.3, 227.3)	0.418	195(167, 238)	188(149, 228)	0.239
Monocytes1, × 10^9^/L, median (IQR)	0.4(0.2, 0.6)	0.4(0.2, 0.5)	0.464	0.4(0.2, 0.6)	0.4(0.2, 0.5)	0.195
Neutrophils1, × 10^9^/L, median (IQR)	7.0(4.6, 9.5)	6.1(3.9, 8.4)	**0.035**	6.6(4.2, 8.8)	6.5(4.7, 9.0)	0.721
Lymphocytes1, × 10^9^/L, median (IQR)	1.2(0.8, 1.7)	1.3(0.7, 2.0)	0.623	1.3(0.9, 1.8)	1.2(0.7, 1.9)	0.297
NLR1, median (IQR)	5.8(2.8, 10.8)	4.7(2.2, 10.1)	0.211	4.9(2.5, 8.9)	5.7(2.4, 12.2)	0.315
Platelet2, × 10^9^/L, median (IQR)	193(158, 235)	184(153, 221.5)	0.252	193(157, 236)	185(154, 220)	0.142
Monocytes2, × 10^9^/L, median (IQR)	0.5(0.2, 0.8)	0.6(0.3, 0.8)	0.164	0.6(0.3, 0.7)	0.5(0.1, 0.8)	0.191
Neutrophils2, × 10^9^/L, median (IQR)	8.2(6.1, 10.4)	9.7(8.3, 11.9)	**< 0.001**	7.5(6.0, 9.6)	9.7(8.4, 11.9)	**< 0.001**
Lymphocytes2, × 10^9^/L, median (IQR)	1.1(0.8, 1.5)	0.8(0.6, 1.0)	**< 0.001**	1.2(0.9, 1.7)	0.8(0.6, 1.0)	**< 0.001**
NLR2, median (IQR)	8.0(5.1, 10.7)	12.2(9.9, 15.8)	**< 0.001**	6.7(4.1, 8.6)	11.7(10.0, 15.5)	**< 0.001**

HT, hemorrhagic transformation; IQR, interquartile range; NIHSS, National Institutes of Health Stroke scale; IVT, intravenous thrombolysis; ASPECTS, Alberta Stroke Program Early Computed Tomography Score; TOAST, Trial of Org 10 172 in acute stroke treatment; ICA, internal carotid artery; MCA, middle cerebral artery; LAA, large-artery atherosclerosis; CE, cardioembolism; TICI, thrombolysis in cerebral infarction; NLR1, neutrophil-to-lymphocyte ratio before thrombectomy; NLR2, neutrophil-to-lymphocyte ratio after thrombectomy immediately. Significant P-values (*p* < 0.05) are marked in bold.

There were no significant differences in demographic characteristics and risk factors between the with-HT group and the without-HT group. The NLR at admission (NLR1) did not differ between patients with and without HT (*p* = 0.211). However, patients who developed HT were prone to higher NLR2 (12.2; IQR, 9.9–15.8 vs 8.0; IQR, 5.1–10.7, *p* < 0.001). According to the unfavorable outcomes, NLR2 was similar to that according to the presence of HT (11.7; IQR, 10.0–15.5 vs. 6.7; IQR, 4.1–8.6, *p* < 0.001).

In comparison to preoperative white blood cell differential values, overall median absolute neutrophil counts increased (9.7; IQR, 8.3–11.9 vs. 6.1; IQR, 3.9–8.4, *p* < 0.001) and lymphocyte counts decreased (0.8; IQR, 0.6–1.0 vs. 1.3; IQR, 0.7–2.0, *p* < 0.001), resulting in an increase in NLR after thrombectomy (8.0; IQR, 5.1–10.7 vs. 5.8; IQR, 2.8–10.8, *p* < 0.001) (Table 2).

**Table 2:** Wilcoxon test results of neutrophil count, lymphocyte count, and neutrophil–lymphocyte ratio (NLR) before and after operation immediately.

	**Pre-operation**	**Post-operation**	***p***
Neutrophils count, × 10^9^/L	6.1(3.9,8.4)	9.7(8.3,11.9)	< .001
Lymphocytes count, × 10^9^/L	1.3(0.7,2.0)	0.8(0.6,1.0)	< .001
NLR	5.8(2.8,10.8)	8.0(5.1,10.7)	0.002

NLR, neutrophil-to-lymphocyte ratio.

### Association of NLR2 with HT and neurological outcomes

Table 3 summarizes the results of the binary logistic regression analysis of HT and neurological outcomes. The NLR2 as a continuous variable was independently associated with a greater risk of HT with an adjusted odds ratio (OR) of 1.31 (95% CI, 1.21–1.42), adjusted for sex and age (model 1), and 1.35 (1.16–1.57), adjusted for occlusion site, cause (TOAST), time from arrival to recanalization, ASPECTS, mTICI, neutrophils1, neutrophils2, lymphocytes2, and NLR2 (model 2), respectively. In addition, the results suggest that longer arrival-recanalization time (adjusted OR 1.01, 95% CI 1.00–1.01; *p* = 0.005) remained a significant risk factor for HT. For the binary logistic regression analysis for unfavorable outcomes, similar associations were found between the NLR2 and unfavorable outcomes. Higher NLR2 (OR 1.85, 95% CI 1.57–2.17; *p* < 0.001) remained significantly associated with unfavorable outcome adjustment for sex, age, occlusion site, NIHSS, ASPECTS, mTICI, neutrophils2, lymphocyte 2, and NLR2 (model 3).

**Table 3:** Associations of NLR2 with HT and unfavorable outcome.

	**HT OR (95%CI)**		**Unfavorable outcome OR (95%CI)**	
	**Model 1**	**Model2**	**Model 1**	**Model3**
NLR2	1.31(1.21, 1.42) ^*^	1.35(1.16, 1.57) ^*^	1.80(1.55, 2.10) ^*^	1.85(1.57, 2.17) ^*^

Model 1 bivariate logistic regression analyses with adjustment for age and sex. Model 2 bivariate logistic regression analyses with adjustment for age, sex, occlusion site, cause (TOAST), time from arrival to recanalization, ASPECTS, mTICI, neutrophils1, neutrophils2, lymphocytes2, and NLR2. Model 3 bivariate logistic regression analyses with adjustment for age, sex, occlusion site, NIHSS, ASPECTS, mTICI, neutrophils2, lymphocytes2, and NLR2. HT hemorrhagic transformation, CI confidence interval, OR odds ratio.

* p < 0.001.

**Figure 2:**
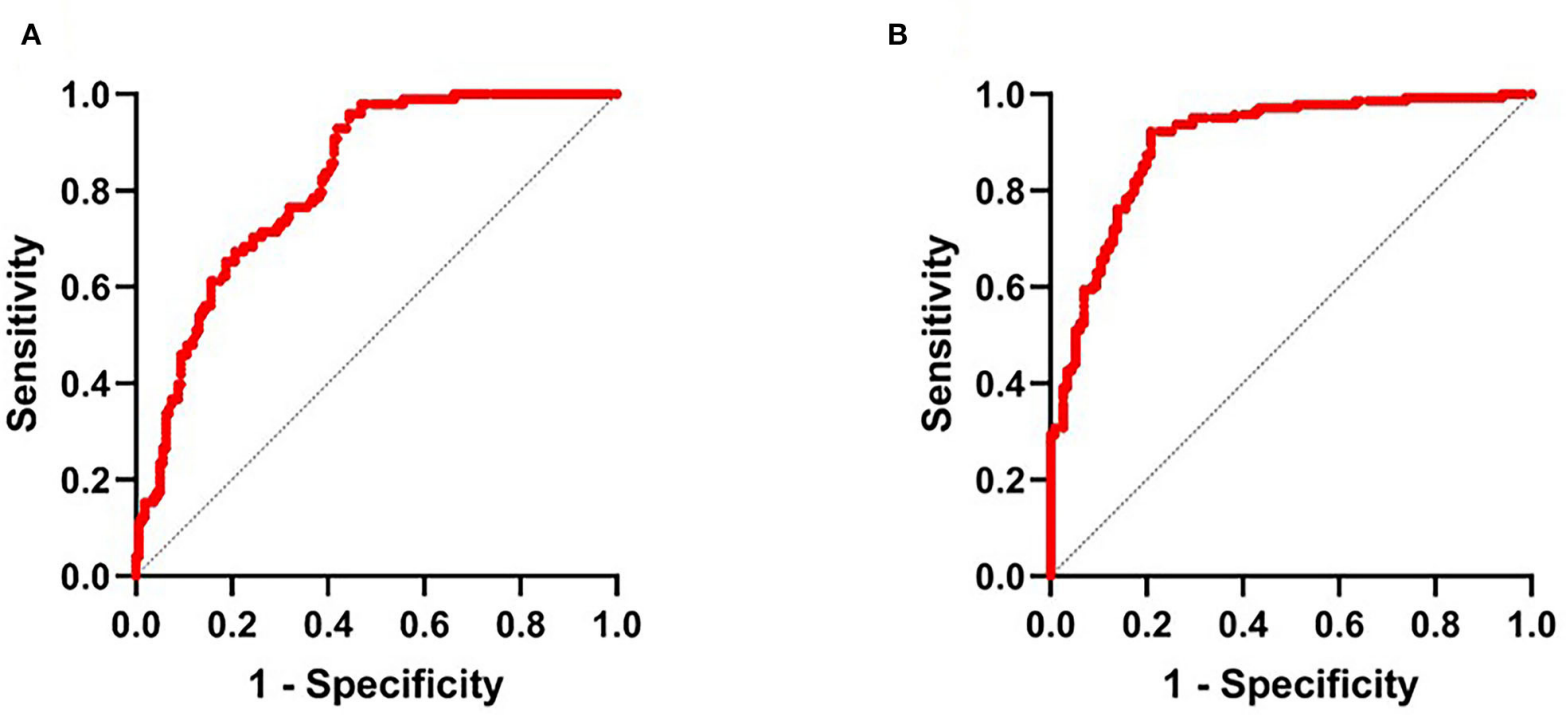
**(A)** Receiver operator characteristic (ROC) curve for NLR2 in the auxiliary diagnosis of HT and **(B)** ROC curve for NLR2 in the auxiliary diagnosis of unfavorable outcome. NLR2, neutrophil-to-lymphocyte ratio after thrombectomy immediately.

Further, Figure 2 showed that the area under the curve (AUCs) of NLR2 for predicting HT after thrombectomy and unfavorable outcomes in patients with anterior circulation AIS was 0.8 (95% CI 0.77–0.87, *p* < 0.001) and 0.9 (95% CI 0.86–0.94, *p* < 0.001). The best cutoff value of NLR2 for predicting HT and unfavorable outcomes after thrombectomy in patients with anterior circulation AIS was 8.4 with a sensitivity of 95.9% and a specificity of 55.6% and 8.8 with a sensitivity of 92.3% and a specificity of 79.1%, respectively.

### Association between NLR2 and subtypes of HT

**Figure 3:**
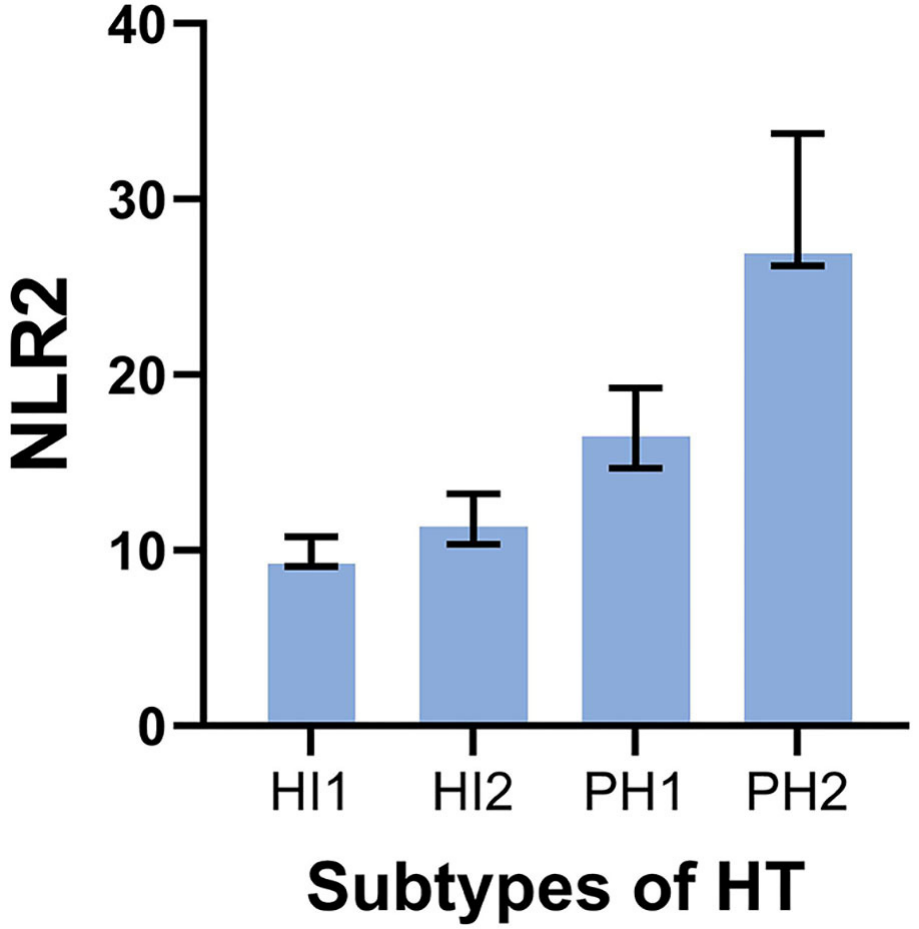
Boxplot of NLR2 and subtypes of HT.

There were 11 patients with PH2 found in the with-HT group, and the results demonstrated that patients with larger hemorrhagic volume had lower lymphocytes 2 after thrombectomy (*p* < 0.001). The association between subtypes of HT and NLR2 is visualized in Figure 3. Other baseline characteristics of four types of HT are shown in Table 4.

**Table 4:** Baseline demographics and clinical characteristics grouped on the basis of types of HT.

**Characteristics**	**HI1**	**HI2**	**PH1**	**PH2**	***p***
**Demographics**					
Age (years)	65 (56.5, 76)	73.5 (62.5, 79)	69.5 (64, 82.5)	73 (57, 86)	0.256
Sex, men, *n* (%)	20 (36.4%)	15 (27.3%)	12 (21.8%)	8 (14.5%)	0.375
**Risk factors**					
Smoking, *n* (%)	11 (40.7%)	7 (25.9%)	6 (22.2%)	3 (11.1%)	0.812
Drinking, *n* (%)	5 (35.7%)	3 (21.4%)	2 (14.3%)	4 (28.6%)	0.206
Hypertension, *n* (%)	19 (30.6%)	24 (38.7%)	13 (21.0%)	6 (9.7%)	0.163
Diabetes, *n* (%)	5 (23.8%)	9 (42.9%)	5 (23.8%)	2 (9.5%)	0.397
Stroke history, *n* (%)	3 (20.0%)	7 (46.7%)	3 (20.0%)	11 (11.2%)	0.390
Atrial fibrillation, *n* (%)	16 (32.0%)	17 (34.0%)	10 (20.0%)	7 (14.0%)	0.615
Coronary heart disease, *n* (%)	16 (34.0%)	18 (38.3%)	9 (19.1%)	4 (8.5%)	0.608
**Clinical data**					
Systolic BP (mmHg)	140 (128.5, 162.5)	142 (124.8, 163.0)	149 (127.5, 181.3)	160 (135, 178)	0.517
Diastolic BP (mmHg)	82 (73.0, 96.5)	78.5 (70.8, 92.3)	83 (78.5, 95.3)	90 (73.0, 103.0)	0.593
Admission NIHSS	14 (10.0, 18.5)	14 (11.3, 16.8)	15 (10.8, 18.3)	18 (14,23)	0.176
Time from onset to recanalization (minutes)	390 (309.5, 611.5)	404.5 (305.5, 609.8)	352.5 (247.5, 459.5)	413 (342.0, 760.0)	0.610
Time from arrival to recanalization (minutes)	218 (175.0, 274.0)	224.5 (163.8, 260.0)	216.5 (183.8, 330.3)	229 (173.0, 291.0)	0.949
ASPECTS	7 (6,8)	7 (6,8)	7 (6, 7.3)	6 (6,7)	0.105
Recanalization (mTICI ≥2b) (n%)	12 (52.2%)	6 (26.1%)	5 (21.7%)	0 (0.0%)	0.119
**Occlusion site**					
ICA	9 (40.9%)	9 (40.9%)	3 (13.6%)	1 (4.5%)	0.390
MCA	16 (30.2%)	19 (35.8%)	10 (18.9%)	8 (15.1%)	
Tandem occlusion	12 (52.2%)	4 (17.4%)	5 (21.7%)	11 (8.7%)	
**Cause (TOAST)**					
LAA (*n*%)	19 (51.4%)	10 (27.0%)	5 (13.5%)	3 (8.1%)	0.253
CE (*n*%)	11 (33.3%)	11 (33.3%)	5 (15.2%)	6 (18.2%)	
Other (*n*%)	7 (25.0%)	11 (39.3%)	8 (28.6%)	2 (7.1%)	
**Laboratory data**					
Blood glucose (mmol/L)	6.4 (5.5, 7.9)	7.5 (5.8, 9.3)	9 (6.2, 11.1)	6.5 (5.4, 9.0)	0.078
Platelet1, × 10^9^/L	193 (157.5, 222.5)	176.5 (115.5, 197.3)	197.5 (173.3, 233.8)	216 (167, 225)	0.079
Monocytes1, × 10^9^/L	1.3 (0.8, 2.2)	1.4 (0.6, 2.0)	1.3 (0.7, 1.9)	1.1 (0.7, 1.4)	0.191
Neutrophils1, × 10^9^/L	5.1 (2.7, 7.9)	5.4 (2.9, 7.1)	7.9 (3.7, 8.9)	8.9 (6.1, 11.1)	0.004
Lymphocytes1, × 10^9^/L	1.3 (0.8, 2.2)	1.4 (0.6, 2.0)	1.3 (0.7, 1.9)	1.1 (0.7, 1.4)	0.664
NLR1	139.1 (102.5, 200.9)	141.4 (108.8, 218.8)	199.2 (104.1, 250.7)	161.3 (120.0, 341.7)	0.063
Platelet2, × 10^9^/L	182 (152.5, 240.0)	166.5 (140.5, 205.8)	194 (151, 213)	199 (127, 218)	0.566
Monocytes2, × 10^9^/L	1.0 (0.9, 1.1)	0.8 (0.6, 1.0)	0.6 (0.6, 0.8)	0.5 (0.3, 0.6)	0.142
Neutrophils2, × 10^9^/L	9.2 (8.0, 10.1)	9.5 (8.0, 11.3)	11.5 (9.3, 12.8)	13.1 (9.8, 15.6)	0.001
Lymphocytes2, × 10^9^/L	1.0 (0.9, 1.1)	0.8 (0.6, 1.0)	0.6 (0.6, 0.8)	0.5 (0.3, 0.6)	< .001
NLR2	203.7 (159.5, 246.5)	228.4 (150.1, 258.0)	270.8 (236.7, 365.0)	332.80 (254.0, 726.7)	< .001

HI, hemorrhagic infarction; PH, parenchymal hematoma; HI1, small patch hemorrhage at the edge of the infarction; HI2, fusion patch hemorrhage in the infarct area, no mass effect; PH1, hematoma < 30% of the infarct area; PH2, the hematoma exceeds 30% of the infarct area.

## Discussion

Our study demonstrates that NLR after thrombectomy immediately predicts hemorrhagic transformation and unfavorable functional neurological outcomes at 3 months post-stroke in consecutive patients with anterior circulation large vessel occlusion stroke. In the present study, we first evaluated the effect of NLR level on the outcome of patients with AIS on admission and re-measured them as soon as possible after thrombectomy. HT after thrombectomy in acute cerebral ischemic infarction is a common complication in patients with acute cerebral infarction, and it is also an important factor for the poor prognosis of cerebral infarction (13). The incidence of HT after cerebral ischemic infarction in Asians is significantly higher than that in Western populations (4). When AIS occurs, peripheral immune and inflammatory cells are activated and enter ischemic brain tissue to play a dual role in the prognosis of cerebral infarction (14).

Inflammation plays an important role in the treatment of ischemic stroke. Microglia, mast cells, and astrocytes are activated immediately after an ischemic event. Neutrophils, cytokines, and chemokines together infiltrate the ischemic area, and these cytokines and chemokines are released into the ischemic area within hours of an ischemic stroke. It has been reported that neutrophils are involved in the formation of NETs (neutrophil extracellular traps) in the whole brain tissue of patients with ischemic stroke (15), and elevated plasma biomarkers of NETs are associated with worse stroke prognosis (16). In the early stage of AIS, neutrophils first migrate and aggregate to vascular lesions, aggravating brain damage by releasing proteases, ROS, and cell adhesion molecules (17); moreover, neutrophils can also infiltrate into the ischemic area, directly destroy the blood–brain barrier (BBB) by enhancing the expression of matrix metalloproteinase 9 (MMP-9), and cause secondary brain injury or hemorrhagic transformation (18). Neutrophil depletion reduces BBB disruption and enhances neovascularization at 14 days (15).

At the same time, some lymphocyte subtypes are involved in multiple inflammatory pathways and may be major brain-protective regulators that reduce infarct volume and improve neurological deficits (19). After ischemic stroke, the number of peripheral T and B lymphocytes is reduced (20). Relative reductions in lymphocytes reflect activation of the cortisol-induced stress response and sympathetic excitation, which increase the production of pro-inflammatory cytokines that aggravate ischemic injury (10). Lower lymphocyte counts are associated with early poor neurological improvement and worse long-term functional outcomes (21). IL-10 is a key neuroprotective cytokine regulating neuroinflammation after stroke. The main sources of brain IL-10 are Treg, Breg, and microglia/monocytes (19). The strategies to increase lymphocyte-derived IL-10 production or therapeutic IL-10 administration have been shown to improve the outcome of stroke (22). With the development of AIS, cerebral ischemia leads to the downregulation of autonomic responses, enhanced neuroinflammatory responses, and increased lymphocyte apoptosis. As a combination of the above 2 biomarkers, NLR is considered to be a good comprehensive indicator of acute inflammation in ischemic stroke patients.

At present, a large number of studies showed that NLR is closely related to cerebrovascular diseases. Wang et al. indicated that high NLR would predict HT and poorer 3-month functional outcomes in patients with AIS undergoing IVT (23). Previous studies showed that, in hemorrhagic stroke, patients with a poor prognosis often have higher NLR, suggesting that it may be a predictor (24). Meanwhile, after adjusting for the NIHSS score at admission, ipsilateral severe intracranial large artery occlusion and atrial fibrillation, the analysis showed that high NLR was a significant predictor of poor prognosis at discharge and 3 months after the onset of stroke (25). A study conducted by Ozgen et al. (26) showed that the higher NLR predicts poor prognosis in patients after mechanical thrombectomy (MT) at 90 days. In Simona's study, they demonstrated that patients who developed sHT had higher NLR at admission (27). This is similar to our study where NLR values were estimated at admission, and the study endpoint was stroke within 24 h of treatment. However, Simons' study included 51 patients with AIS with a median age of 67 years and sHT occurred in 10 (19.6%) patients. This sample size was smaller than ours. This would explains why our NLR at admission was not statistically significant with postoperative HT. Mauro found that, among patients with ischemic stroke who underwent Endovascular treatment (EVT) and reached successful recanalization, those with higher SIRI (Systemic Inflammatory Response Index) at admission were at increased risk of poor 3-month functional outcome (28). We studied the relationship between inflammatory biomarkers and functional outcomes after EVT. Their total white blood cells were collected from admission blood work within 24 h after stroke onset. While in our study, venous blood samples were obtained before and after thrombectomy immediately. In addition, patients with a pre-stroke mRS score >2 and patients who did not have admission laboratory values and/or 3-month outcome were excluded in Mauro's study. In our study, we excluded the patients who have malignant tumors, blood system diseases, liver, and kidney failure. Pektezel et al. reported that, as a marker of stroke-associated acute stress response, the increased NLR is an epiphenomenon of poor prognosis during the first 24 h. However, pretreatment NLR values have no importance in predicting IV tPA response (29). Unlike other studies, we investigated more detailed monitoring metrics, including platelet counts, neutrophil counts, lymphocyte counts, and NLR in patients at first admission and after thrombectomy immediately. Increased neutrophil/lymphocyte ratio after thrombectomy immediately is consistent with Pektezel's study. Further, our study proved that, among patients with anterior circulation AIS who underwent thrombectomy, the median postoperative remeasured NLR2 (7.99) was higher in patients with postoperative intracerebral hemorrhagic transformation. Notably, most of the elevated NLR occurred after thrombectomy. Therefore, it is more important in the follow-up observation.

After that, we divided HT into HI and PH according to ESCASSIII. Our results found that, with the exaltation of NLR2, there are differences between the subtypes of HT and the mechanism possibly because the occurrence of HT is mainly related to the destruction of the BBB, and the severity of HT may be mainly attributed to endothelial injury (30). Studies showed that endothelial cell dysfunction can cause blood–cerebrospinal fluid barrier damage and microcirculation disorders, resulting in impaired neurological function and induction of END (early neurological deterioration). At the same time, neutrophils effectively activate NOX2 primarily through binding to certain members of G protein-coupled receptors (GPCRs) (31), thereby generating ROS, which in turn destroy vascular endothelial cells.

However, this study has certain limitations, as it is a retrospective study, so there may be selection bias. In our study, the small size weakens the statistical strength of our conclusions. For example, our research showed that the admission time was not related to a bad outcome and patients with ischemic stroke undergoing EVT and reaching successful recanalization who had higher inflammatory biomarkers at admission were not at increased risk of poor 3-month functional outcome, which conflicts with previous research. Future studies will include a broader set of AIS thrombectomy cases to further elucidate the association of NLR with hemorrhagic severity on admission and after thrombectomy. Nonetheless, our study provides valuable findings to some extent. First, NLR does not increase in the hyperacute phase, especially before thrombectomy. Therefore, NLR cannot be used as a criterion for evaluating whether a patient needs thrombectomy or not. Second, NLR2 is a sensitive biomarker for the development of HT after thrombectomy and neurological outcomes in patients with anterior circulation AIS and predicts the association of NLR2 with bleeding volume. Last but not least, it is unclear whether this phenomenon is a cause or a consequence of clinical deterioration. However, it can still be used as an index for monitoring treatment.

In conclusion, NLR2 is an important predictor of hemorrhagic transformation and neurological outcomes in patients with anterior circulation AIS after thrombectomy. In future, incorporating NLR2 into the risk assessment system will allow physicians to identify patients at high risk for HT after thrombectomy and implement appropriate management to reduce medical stress.

## Data availability statement

The original contributions presented in the study are included in the article/supplementary material, further inquiries can be directed to the corresponding author.

## Ethics statement

The studies involving human participants were reviewed and approved by the Ethics Committee of Shanghai Ninth People's Hospital, Shanghai Jiao Tong University School of Medicine (No. SH9H-2020-T390-2). Written informed consent for participation was not required for this study in accordance with the national legislation and the institutional requirements.

## Author contributions

J-RL contributed to the conception and design of the study. S-JL and S-SC contributed to drafting the text and preparing the figures. All authors contributed to the acquisition and analysis of data.

## Funding

This research was supported by grants from 200 talent projects from Shanghai Municipal Education Commission—Gaofeng Clinical Medicine Grant Support (No. 20161422 to J-RL) and the Natural Science Foundation Project from the Shanghai Municipal Science and Technology Commission (No. 22ZR1436900 to J-RL).

## Conflict of interest

The authors declare that the research was conducted in the absence of any commercial or financial relationships that could be construed as a potential conflict of interest.

## Publisher's note

All claims expressed in this article are solely those of the authors and do not necessarily represent those of their affiliated organizations, or those of the publisher, the editors and the reviewers. Any product that may be evaluated in this article, or claim that may be made by its manufacturer, is not guaranteed or endorsed by the publisher.
